# Acute aortic dissection in pregnancy management with the fetus remaining in situ

**DOI:** 10.1186/1749-8090-8-S1-O255

**Published:** 2013-09-11

**Authors:** DC Ferreira, IM Abreu, J Honório, PN Sampaio, F Binello, D Bastos, J Pierre, IH Carvalho, U Alves, A Bosiger, PMS Pereira Filho, DH Silva, O Souza Neto

**Affiliations:** 1 Department of Cardiovascular Surgery, Hospital Federal dos Servidores, Rio de Janeiro, Brazil

## Background

Acute aortic dissection is one of the most dreaded clinical conditions during pregnancy. The limited experience reported in the literature does not allow the determination of guidelines for surgical management of aortic dissection in these cases. In this case presentation we successfully treated a 27-year-old woman with Marfan syndrome in the 36th week of pregnancy with acute type A aortic dissection, who underwent aortic repair with the fetus remaining in situ.

## Methods

After a review of the data reported in the literature, we present this case of acute aortic dissection in a pregnancy woman with Marfan syndrome and discuss some new perspective of surgical management and maternal-fetal outcome considering the peculiarities of this disease's manifestation.

## Results

Surgery for acute aortic dissection during pregnancy has been described by other investigators and, in most cases; the fetal outcome was relatively poor. The review of the data suggests that, in cases of fetal maturity, Cesarean section should be performed before or in combination with aortic repair. However, the appropriate surgical management with an immature fetus in utero remains unclear. The cardiopulmonary bypass (CPB) with the fetus in utero may itself represent a risk factor, as already demonstrated by studies that hypothermia contributes for a worse prognosis. We present a case of a 36th weeks pregnancy patient with Marfan syndrome who had acute type A Aortic dissection and underwent operative repair with modified Bentall de Bono technique with the fetus remaining in situ, where during CPB was used high-flow, high pressure and mild hypothermia. The maternal-fetal outcome was excellent and an elective Cesarean of the fetus could be done one week later the aortic repair when the clinical conditions of the mother was completely stabilized and the fetus presented better maturity.

## Conclusions

Despite the controversies of surgical management of aortic dissection during pregnancy because the lack of data, it seems to be possible as established in this case presentation, with the development of CPB and surgical techniques, perform the aortic repair with the fetus remaining in situ.

